# Microtubule Regulation in Plants: From Morphological Development to Stress Adaptation

**DOI:** 10.3390/biom13040627

**Published:** 2023-03-30

**Authors:** An-Shan Hsiao, Ji-Ying Huang

**Affiliations:** 1 Department of Biochemistry and Metabolism, John Innes Centre, Norwich Research Park, Norwich NR4 7UH, UK; 2 Cell Biology Core Lab, Institute of Plant and Microbial Biology, Academia Sinica, Taipei 115, Taiwan; qqhair@gate.sinica.edu.tw

**Keywords:** microtubules, microtubule-associated proteins, development, patterning, morphogenesis, stress adaptation

## Abstract

Microtubules (MTs) are essential elements of the eukaryotic cytoskeleton and are critical for various cell functions. During cell division, plant MTs form highly ordered structures, and cortical MTs guide the cell wall cellulose patterns and thus control cell size and shape. Both are important for morphological development and for adjusting plant growth and plasticity under environmental challenges for stress adaptation. Various MT regulators control the dynamics and organization of MTs in diverse cellular processes and response to developmental and environmental cues. This article summarizes the recent progress in plant MT studies from morphological development to stress responses, discusses the latest techniques applied, and encourages more research into plant MT regulation.

## 1. Introduction

Microtubules (MTs) are highly conserved cytoskeletal structures in both plant and mammal cells [1,2]. Like mammal MTs, plant MTs consist of α- and β-tubulin subunits [3,4], and some tubulin isoforms are expressed in specialized cells or tissues during development [5,6,7,8]. The formation of α/β-tubulin heterodimers needs a large chaperone complex and guanosine 5′-triphosphate (GTP) [9,10]. Hence, recombinant tubulins cannot be efficiently produced in *Escherichia coli* because of the lack of a proper protein folding machinery in prokaryotes [11]. The longitudinal head-to-tail interactions between α/β-tubulin heterodimers via GTP hydrolysis to guanosine diphosphate build up the basic units of MTs, protofilaments [12,13]. The GTP cap at the plus end ensures MT growth, while the loss of the GTP cap results in MT shrinkage. The co-existence of growing and shrinking MTs driven by the restoration and hydrolysis of GTP was proposed as a “dynamic instability” model based on the observation of in vitro-reconstituted MTs [14]. The dynamic behaviour of MTs is thought to be an intrinsic property, as demonstrated by a MT polymerization experiment conducted from purified tubulin without external factors [15].

MT polymerization can occur spontaneously in vitro without any pre-formed templates when sufficiently high concentrations of purified tubulins are warmed up in the presence of GTP [16]. However, in cells, tubulin molecules tend to form a nucleation seed for efficiently initiating polymer growth and the construction of dynamic polar MTs under spatial and temporal control [17,18]. The evolutionarily conserved MT nucleating template is known as the γ-tubulin-containing ring complex (γ-TuRC) [19,20,21]. It initiates MT nucleation at a particular subcellular location, primarily regulated by Augmin [22,23,24]. Katanin internally breaks MTs dependent on adenosine 5′-triphosphate (ATP), particularly at MT crossover positions, where the detached daughter MTs can translocate via treadmilling to form new configurations of MT arrays [25,26,27,28,29]. Thus, both γ-TuRC and katanin are thought to be central components in synthesizing new treadmilling MTs at the plant cell cortex [25,30]. The dynamic nature enables MTs to alter their organization in response to internal and external signals for the needs of the cell, and it is regulated by various proteins [31,32]. 

Eukaryotes have conserved MT-associated proteins (MAPs) that bind along the MT lattice and have stabilizing or destabilizing effects on MT assembly [32,33,34]. However, plants possess a set of MAPs specific to plant morphology and physiology [35,36,37]. Conventional MAPs include motor proteins such as kinesins that utilize MTs as tracks to transport cargo and structural MAPs or severing proteins such as MAP65 and katanin (with the catalytic subunit p60 and a regulatory subunit p80) involved in MT organization via binding, bundling, or cleavage of MTs. MTs plus tip-associated proteins, such as cytoplasmic linker-associated proteins (CLASPs), regulate MT dynamics via their binding and interactions at the plus-end of growing MTs [13,36,38,39,40,41,42,43]. 

MTs arise from centrosomes in animal cells [44], but MTs in acentrosomal plant cells are thought to self-organize into structured arrays [45]. The plant-specific structures (i.e., cell wall and stomata) and the sessile nature of land plants lead to distinct MT regulation, affecting plant growth, development, and stress adaptation. This article summarizes the latest research progress (mostly after 2018) in plant MT regulation from the cellular to organism level and discusses cell division and intracellular trafficking, morphogenesis and development, as well as stress responses. By introducing the latest technologies in studying plant MTs, we aim to encourage more research to discover the fundamental rules governing the dynamics and molecular mechanism of how cells specify the functional MT array patterns under various circumstances.

## 2. MTs in Plant Cell Division

**Figure 1:**
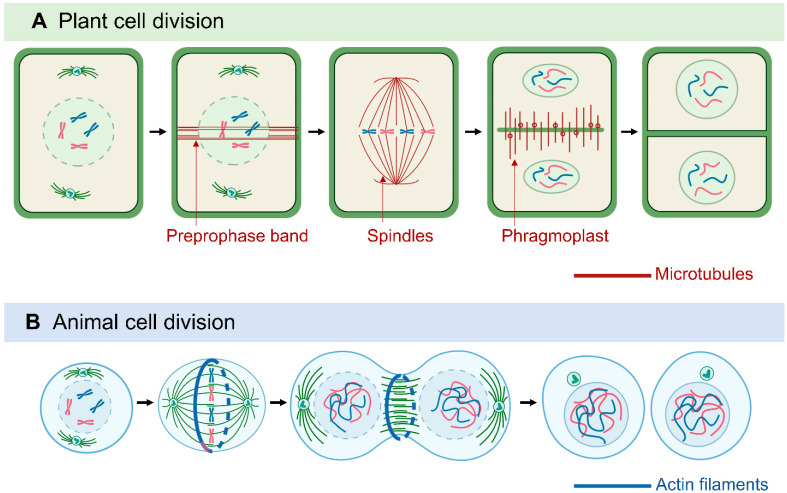
Comparison of plant and animal cell division. Plant cell division (**A**) is characterized by microtubule (MT)-based structures: the preprophase band, the acentrosomal mitotic spindle, and the phragmoplast. In animal cell cytokinesis (**B**), the contractile ring pinches the cell into two daughter cells, whereas plant phragmoplasts extend and guide vesicle fusion to generate the cell plate.

Sessile plants cannot move as quickly as multicellular animals to escape environmental challenges. Thus, besides forming organs and various cell types for morphological development, cell division in plants is also important for adaptation to environmental conditions: adjusting growth under stress by enhancing, reducing, and redirecting cell growth [46]. In dividing plant cells, MTs form distinct structures, including the preprophase band (PPB), the acentrosomal mitotic spindle, and the phragmoplast [47,48,49] (Figure 1). The PPB, which is a plant-specific cortical MT ring, marks the orientation of the cell division plane and determines the spindle positioning in metaphase [50,51,52]. The PPB tunes the orientation of spindles in a mode similar to that of centrioles and astral MTs in animal cells, which implies the importance of spindle orientation [51,53].

MT-based spindles separate chromosomes during mitosis [54]. Most animal spindle-assembly factors are well conserved in plants, but plants lack two major elements: centrosomal components and the cytoplasmic dynein complex [48]. Animal spindle MTs nucleate from centrosomes [55], whereas plant MTs appear to nucleate from the nuclear envelope surface [56]. In animals and fungi, cytoplasmic dynein is a processive minus-end-directed motor that pulls on astral MTs from the cell cortex for efficient and accurate spindle assembly and positioning [57]. In contrast, plants lack cytoplasmic dynein but contain many minus-end–directed kinesin-14 proteins, which were thought to be involved in the sliding of anti-parallel microtubules throughout mitosis [46,58,59,60]. Kinesins with a calponin homology domain (KCH) are a distinguished subclass of kinesin-14 found only in Plantae [61]. Rice OsKCH2 exhibits processive minus-end-directed motility on MTs to potentially compensate for the loss of dynein [62], whereas moss KCH drives MT-based nuclear transport reminiscent of animal dynein [63]. In animals, plus-end–directed kinesin-5 and kinesin-12 facilitate the spindle arrangement [64,65] and it seems to be conserved in plants, as shown by Arabidopsis KINESIN-12E controlling spindle MT organization and size during mitosis [66]. MTs are protein–protein interaction sites for the spindle assembly checkpoint (SAC) protein complex and signaling network. Plants have conserved the SAC network with some variations from animals and yeast [67]. The dissection of proteins associated with SAC led to the discovery of novel aspects of plant SAC regulation [68,69], which may be relevant for plant breeding studies because ploidy alternations likely rely on SAC properties. 

Another plant-specific MT machinery, the phragmoplast, is formed between the reconstituting daughter nuclei at the end of telophase, as a hallmark of cytokinesis [70]. The phragmoplast expands and transports Golgi-derived vesicles containing building materials to facilitate the construction of the cell plate. The assembly, crosslinking, and turnover of phragmoplast MTs are regulated by various MAPs, kinesin motors, and regulatory enzymes [49,53,70,71,72]. Among them, plant-specific Cortical MT Disordering 4 tethers the conserved MT-severing protein katanin to facilitate phragmoplast expansion and accelerate cytokinesis [73]. Cytokinesis-specific MAP65-3 plays primary roles in phragmoplast integrity and efficient cell plate formation [74,75,76]. Phragmoplast dynamics during cytokinesis is closely related to the phosphorylation of MAP65-3, regulated by mitogen-activated protein kinase 4 and aurora kinase [77,78,79]. As a positive regulation mechanism, benzimidazole-3 proteins interact with MAP65-3 and promote MT bundling for phragmoplast expansion [80]. The MT motor protein KINESIN12 is critical for maintaining MT plus-ends in the phragmoplast midzone. Indeed, the Arabidopsis double-mutant pok1/pok2 (two kinesin-12 orthologs) revealed chaotic division sites and a slower phragmoplast expansion rate compared to the wild-type [81,82]. Overall, because they lack structurally defined centrosomes but have flexible and distributed PPB and phragmoplasts, plant cells can assemble bipolar spindles and determine the division plane with a great deal of plasticity, thereby compensating for the restraints in cell movement caused by the stiff plant cell wall [46,83]. 

## 3. Cortical MTs in Intra- and Intercellular Trafficking 

Cortical MTs are tightly anchored to the plasma membrane and interact with plasmodesmata and several endomembrane systems. Therefore, they are involved in intra- and intercellular transportation [84,85]. Recent studies showed that MT-dependent homeostasis of the *trans*-Golgi network (TGN) is regulated by TGNap1 [86], whereas MTs and kinesins participate in vacuole trafficking and endoplasmic reticulum (ER) movement as revealed by modified transport to the vacuole proteins [87,88]. MTs are a scaffold for stromule extension, branching, and kinking [89,90] (see Section 7) and are also involved in nuclear transport to regulate tip cell growth in moss and Arabidopsis root hairs [63,91]. MTs have a role in the intercellular movement as shown by KINESIN G facilitating the cell-to-cell movement of short-root along MTs [92]. Additionally, mobile microRNAs are regulated by MTs and KATANIN1 via ARGONUATE1 of the RNA-induced silencing complex by an unknown mechanism to exit the cell [93]. 

## 4. MTs in Morphological Development 

MTs are essential in cell wall formation by guiding the movement of cellulose synthase complexes on the plasma membrane. Therefore, they function as templates for the deposition of cell wall cellulose [94,95,96]. The orientation of cellulose microfibrils affects cell wall stiffness and cell growth direction, and in turn, the mechanical stress generated by the growing tissue orients the MTs, thus forming a feedback loop [97]. Perturbation of MTs often leads to changes in cell wall composition and cell stiffness, thus affecting cell expansion and plant architecture [98]. Hence, plant cell morphogenesis requires a dynamic interplay between cell expansion and MT-templated cell wall construction [37].

### 4.1. Seed Germination

When dormant seeds are imbibed, MTs appear as thick bundles in random orientations [99]. Dormancy release in the embryo triggers MT self-organization and alignment with tensile stress before germination and anisotropic growth [99]. Various MAPs and hormone signaling are involved in seed germination. Wave-Dampened2-Like 4 modulates auxin distribution to promote apical hook opening in Arabidopsis [100]. Additionally, the elongated hypocotyl cells create a variety of MT array patterns with differing degrees of polymer coalignment and orientation to the cell growth axis [101]. These patterns are regulated by light and ethylene signaling via Armadillo Repeat Kinesin 2 and Microtubule-Destabilizing Protein 60 [102,103].

### 4.2. Root

Recent studies have highlighted the importance of MTs in root morphogenesis and root hair and apical meristem growth by investigating the MAPs TANGLED 1, Auxin-induced-in-roots 9, CLASP, and Rho of Plant interactors and extensive study of KATANIN 1 [104,105,106,107]. Functioning in nutrient and water uptake, the initiation and directional growth of lateral roots are largely regulated by the dynamics of MT networks [108,109]. MAP70-5 is necessary for spatially defined MT organization and endodermis remodeling during lateral root morphogenesis [110], whereas MT reorganization via MT-stabilizing protein TPX2-LIKE5 during lateral root initiation is negatively regulated by Elongated Hypocotyl 5 [111]. Similarly, rhizoid tip growth in the basal land plant *Marchantia polymorpha* is controlled by the MT organization with different MAPs from flower plants: Wave-Dampened 2-Like [112] and MpNEK1 [113]. The plant hormones auxin and brassinosteroid (BR) regulate root growth via dynamic MT organization as shown by FERONIA mediating root nutation growth by affecting MT polymerization and polar auxin transport [114]. Additionally, Tetratricopeptide-repeat Thioredoxin-Like 3 interacts with MTs and participates in BR signaling during root system morphogenesis [115].

### 4.3. Leaf

Correct timing is needed to orchestrate MT dynamics and cell wall biogenesis during leaf development [116]. Leaf shape depends on cortical MT-mediated cellulose deposition along the adaxial–abaxial axis in the internal cell wall [117]. MTs may act as sensors or as part of sensing mechanisms for mechanical stress [118]. Mechanical stress promotes the MT response to stress by increasing KATANIN severing activity [119] and initiates and sustains the morphogenesis of wavy leaf epidermal cells [120]. Cortical MTs are thought to align with maximal tension, whereas adjustment of MT stabilization with a tension–adhesion feedback loop with disturbance of this linkage to the wall could provide “noise,” allowing for greater adaptive response to new inputs, both mechanical and developmental [121,122]. MT dynamics during pavement cell morphogenesis are regulated by BR and auxin [123,124] as well as various MAPs such as IPGA1-ANGUSTIFOLIA [125], IQ67 DOMAIN 5 (IQD5) [126], KATANIN and CLASP [127], and basic proline-rich protein [128]. 

### 4.4. Xylem

Cortical MT alignment regulates distinct deposition patterns of the secondary cell wall in xylem vessels [129]. Spatiotemporal control of MT nucleation and KATANIN-regulated MT band formation are critical for xylem wall patterning [130,131]. Xylem pit formation requires mutually exclusive plasma membrane tethering by the Microtubule Depletion Domain1–Kinesin13A complex and IQD13 for MT depolymerization and stabilization [132,133,134,135,136]. Recently, the MT regulator MAP20 was reported to be involved in metaxylem pit development [137] and Cortical MT Disordering1 for proper deposition patterns in metaxylem vessels [138]. 

### 4.5. Flower

Cortical MTs play important roles in plant cell polar growth/expansion and have been reported to guide the growth and shape of sepals and petals [139]. MAPs were found to modulate cortical MT arrangement in flower organ growth and fertility: Increased Petal Growth Anisotropy 1, QWRF1, and QWRF2 affect MT organization and stability to regulate petal growth anisotropy [140,141], and KATANIN 1 mediates MT organization to regulate petal conical cell shape [142,143] and the pollen tube path [144]. KATANIN 1-mediated MT severing is critical for stamen filament development, as shown by the crosstalk between MT severing and BR signaling [145].

### 4.6. Fruit, Grain, and Seed

Cortical MT arrays play a critical role in plant cell shape determination [146]. Controlling the shape and architecture of a plant could help improve agricultural yields. The plant-specific IQD family emerged as regulators of fruit shape and grain size in tomatoes [147] and rice [148]. By interacting with MT arrays, TONNEAU 1-recruiting motif (TRM) protein Grain Weight 7 controls grain dimension traits in wheat [149], whereas Reducing Plant Height 1 controls plant and ear height in maize [150]. The plant seed coat protects the embryo and transmits information regarding the external environment [151]. TRM4 and IQD9 maintain cortical MT organization to orchestrate cellulose patterning in Arabidopsis seed mucilage [152,153]. MTs are important for the development and diameter determination of cotton fiber [154], an extension of seed coat [155], as shown by a recent finding that GhMAP20L5 is involved in cotton fiber elongation by interacting with GhTUB13 [156]. 

## 5. MTs in Abiotic Stress Responses

### 5.1. Temperature

Sessile plants have evolved specific mechanisms to perceive environmental stresses and generate appropriate responses. The plant cytoskeleton plays central roles in stress-induced signaling pathways, either as a direct target or a signal transducer [157]. Cell wall remodeling and MT rearrangements are essential for plants to adapt to growth and development in response to environmental stresses [158]. Temperature stresses such as coldness and heat restrict plant growth and development and thus affect crop production. In grapevine, cold-induced disassembly of MTs is regulated by a key transcription factor, Cold Box Factor 4 [159], and MTs were proposed to serve as modulators of cold sensitivity by mediating the transportation of cold signaling vesicles into the plasma membrane [160,161]. Rice MT motor Dual Localisation Kinesin enters the nucleus under cold stress, but whether MTs are involved in cold-induced transcriptional regulation needs further investigation [162]. Recent studies showed that phospholipase D δ regulated reactive oxygen species-mediated MT organization and stomatal movement upon heat shock, and heat shock protein 70-3 bound to MTs and interacted with phospholipase D δ to stabilize cortical MTs upon heat stress [163,164], which suggests the importance of controlling MT dynamics in the heat stress response.

### 5.2. Salinity

At the cellular level, salinity stress induces rapid depolymerization of cortical MTs and further reassembly into altered MT networks [165,166]. Companion of cellulose synthase (CC) proteins control MT reassembly and support cellulose synthase activity to sustain plant growth under saline conditions [167,168]. A recent proteomic analysis revealed the upregulation of MT proteins (TUB3, TUB4, and TUB9) in salt-adapted Arabidopsis. Loss of function of TUB4 enhanced salinity tolerance, and mutation in TUB9 caused hypersensitivity [169], but the exact mechanism still awaits discovery. Various MAPs play a significant role in mediating dynamic MT changes under salinity; these include Arabidopsis KATANIN 1 [170], MAP65 [171], SPIRAL 2-LIKE [172], and rice microtubule-associated RING finger protein 1 [173]. Moreover, ethylene signaling regulates MT reassembly in response to salt stress in Arabidopsis [174]. Analysis of the rice OsTUB1–Kinesin13A complex revealed the function of tubulin and kinesin in regulating MT organization and ionic homeostasis to increase the survival of rice plants under salt stress, thus providing novel genes for salt-insensitive rice breeding in areas with high soil salinity [175]. 

### 5.3. Drought

The precise regulation of stomatal movement is critical for plant adaptation to drought stress [176]. The disassembly of MTs is critical for stomatal closure in response to abscisic acid (ABA) and drought stress [177]. E3 ligases were recently reported to participate in ABA-mediated MT depolymerization, stomatal closure, and the drought stress response; these ligases include MT-related E3 ligase 57 [178] and JAV1-associated ubiquitin ligase 1 [179]. SUN-interacting nuclear envelope protein 1 (SINE1) and SINE2 as well as SPIRAL1 were also found involved in MT reorganization during ABA-induced stomatal closure [180,181], and tubulin perturbation affected guard cell behavior, delaying drought-induced stomatal closure and clustering [182,183]. 

## 6. Protein Disorder Regulates MTs

Intrinsically disordered proteins (IDPs) are a group of functional proteins without defined 3D structures. They can adopt various conformations when binding to different partners and serve as interaction specialists in cellular signaling and the regulation of macromolecular machine assembly [184,185,186]. The roles of IDPs in regulating adaptive responses and the dynamics of the cytoskeletal systems have been summarized in animal cells [187]. Both MT-binding MAP2 and Tau proteins are intrinsically disordered [188]. The disordered regions of Tau are important for MT-binding and pathological aggregation, which cause neurodegeneration in Alzheimer’s disease and related dementias [189]. In plants, Arabidopsis CC1 controls MT bundling and dynamics to sustain plant growth under salt stress via its intrinsically disordered N-terminus through a Tau-like mechanism [168]. Recently, a rice disordered repetitive proline-rich protein was found as a novel mechanism of a plant IDP controlling highly ordered actin filaments and MTs to adapt root growth under water deficit [190]. Even though IDPs can control MT dynamics in both mammals and plants, the results are very different: neuropathology in mammals and stress adaptation in plants. In comparison to well-understood mammal protein disorders in MT regulation and function in neurodegenerative diseases, plant research about how protein disorders regulate MTs is lagging. Given that the disordered nature of IDPs plays regulatory roles in highly ordered MTs, this field needs more attention.

## 7. MTs in Biotic Stress Responses

Upon pathogen attack, the plant cytoskeleton undergoes rapid remodeling to coordinate the movement of intracellular organelles and the formation of immune microdomain complexes as well as the transportation of defense compounds and the turnover of recognizing receptors [191]. Although actin remodeling is thought to play an essential role during plant innate immunity, recent studies have highlighted that immunity-related protein secretion and cell wall-based defense depend on MT-based transport [192]. The role of MTs in intracellular trafficking and secretory pathways may have a positive or negative impact on plant–pathogen interactions. Cortical MT-associated ER sites are essential for the replication and cell-to-cell spread of the tobacco mosaic virus [193]. Chloroplast stromules facilitate the transport of pro-defense signals into the nucleus during innate immunity [194], and stromule extensions through the ER are directed by MTs [195,196], which implies the importance of MTs in chloroplast–nucleus communication. Additionally, MTs but not actin cytoskeleton exclusively control the dynamics of aquaporin AtPIP2;1 during stomatal closure in response to flg22 [197], which suggests the unique function of MTs rather than actin filaments. 

### 7.1. Plant–Pathogen Interaction

**Figure 2:**
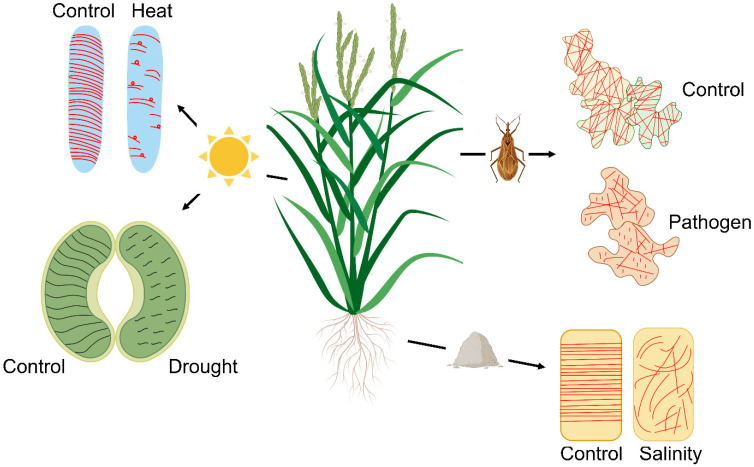
MT networks in response to environmental stresses. Environmental stresses cause the change in MT networks in plant cells. Insets illustrate MT network changes after pathogen infection and treatments for heat, salinity, and drought stress.

Pathogens produce effectors targeting the cytoskeleton to achieve pathogenicity [191]. By interacting with MAPs, pathogens can control the MT network in the host cells [195,198,199]. For example, the *Pseudomonas syringae pv. tomato* (*Pto*) effector HopE1 dissociates MAP65-1 from MTs to inhibit protein secretion and cell wall-based extracellular immunity to benefit pathogen *Pto* growth [199]. MAPs are targets of bacterial pathogen effectors, and other pathogens also co-opt MAPs for facilitating pathogenic penetration into host cells [200]. The effector protein ROP-Interactive Peptide 1 of the powdery mildew fungus *Blumeria graminis f.sp. hordei* (*Bgh*) is involved in *Bgh* virulence and also destabilizes cortical MTs in barley cells [201]. The nematode effector GpSPRY-414-2 targets MTs to facilitate infection [202]. By targeting MAPs, pathogens may weaken the host defense responses regulated by MTs, such as the delivery of antimicrobial molecules to the sites of infection, and thus increase pathogen growth/proliferation [203]. However, the anisotropic patterning of cortical MTs is required for regulating immunity-related genes in distal cells, as revealed by cortical MT-mediated mechanotransduction of pathogen-derived cues facilitating disease resistance caused by the fungus *Sclerotinia sclerotiorum* [204]. Figure 2 shows examples of MT network changes in response to environmental stresses.

### 7.2. Symbiosis

Besides non-beneficial plant–pathogen interactions, MTs also function in beneficial symbiosis processes between plants and symbiotic microorganisms [205]. Active cytoskeletal rearrangements are required at different stages of nodule development, from root-hair curling for entrapment of rhizobia to infection thread growth [206,207,208]. Endoplasmic MTs appear to support infection thread growth, infection droplet formation, and bacterial release into the host cytoplasm in nodules of legume species, whereas irregular cortical MT arrangements provide a possibility for isodiametric cell growth that allows a notable increase in cell size for hosting numerous symbiosomes [209]. The reorganization of MT networks during symbiosis is regulated by MAPs. In Medicago, Developmentally regulated plasma membrane polypeptides trigger MT fragmentation in symbiosis-specific membrane nanodomains to establish symbiotic associations [210]. TPXL and MAP65, together with AURORA 1, form a mitotic module to regulate MT functions for supporting the infection-thread formation of rhizobia during legume endosymbiosis [211]. Rhizobia hijack a plant-specific kinesin motor to crosslink MTs with actin filaments for controlling central vacuole formation to achieve symbiosome development and nitrogen fixation [212]. Substantial MT remodeling has been observed in arbuscule-containing cells [213,214]. A recent report revealed that a *Solanaceae*-specific MAP, tomato similar to SB401, is involved in MT bundling and rearrangements occurring in arbuscule-containing cells, which are required for proper arbuscule development and activity [215].

## 8. Key Questions in MT Regulation

Several key questions in plant MT regulation remain to be answered. Compared to fungi and animals, factors that regulate spindle MT remodeling in anaphase are largely unknown in plants. What are the novel regulatory proteins and their fine-tuned features associated with MT dynamics during cell division as well as the impact on plant polyploidization? Plants perceive various intrinsic and extrinsic mechanical stress signals during developmental processes. How is the stress in the cell wall transferred into cortical MTs, and what is the role of MTs as a stress sensor in the regulation of plant cell growth? When plants encounter environmental challenges, MTs are involved in various abiotic stress tolerance responses. How do IDPs regulate plant MTs for stress tolerance? What are the roles of MTs in stress sensing and signaling and which MAPs participate in stress adaptation? 

## 9. New Techniques for Studying Plant MTs 

To answer the aforementioned key questions, here we discuss several examples of new technology developed recently for plant MT research in silico, in vitro, and in vivo.

### 9.1. In Silico

Studies describing cytoskeleton dynamics rely on qualitative/quantitative analyses of cytoskeleton images. A new filament segregation algorithm, Implicit Laplacian of Enhanced Edge (ILEE), was created to provide accurate and robust analyses of cytoskeleton 2D and 3D images, thus eliminating the traditional limitation and user bias of the approach involving manual global thresholding. Indeed, ILEE can process plant MT images with satisfying performance [216]. Because cortical MT organization is a determining factor for division plane formation [53], a computer simulation method was developed to capture the effects of cell geometry on MT organization. Additionally, this method describes the stabilization of MTs at cell edges and includes the effects of polar auxin signaling on local MT stability to improve division plane prediction [217]. 

### 9.2. In Vitro

In vitro studies are essential for the biochemical characterization of interactions between various MT regulators and MTs. Even though eukaryotic tubulin proteins are highly conserved and the assembly and disassembly mechanisms of MT are thought to be similar, the kinetics of MT dynamic instability differs for animal and plant tubulins [218]. Interactions with MT-interacting proteins may differ for tubulins isolated from different organisms [219]. Thus, plant tubulin but not porcine brain tubulin must be used for in vitro studies of plant MT regulation. A TOG-based column (named after the human MT regulator tumor overexpressed gene [TOG]) was used to purify assembly-competent tubulin from fungal, animal, and algae sources [220]. The breakthrough technology also allowed for purifying wild-type and recombinant functional tubulin from Arabidopsis and tobacco [221,222]. This will facilitate in vitro functional studies of tubulin mutants, tubulin posttranslational modifications, and interactions with various plant MT regulators and motor molecules, for example [221]. 

### 9.3. In Vivo

Genetically encoded MT markers in the dicot model plant Arabidopsis have been well developed [223]. However, cytoskeleton fluorescent marker lines were recently generated in the monocot model plant rice [224]. In contrast to Arabidopsis and maize [150,223], the use of tubulin subunits cannot label MTs well in rice [224]. Conversely, the use of the mammalian MAP4 [225] in the mScarlet-MAP4 rice line allowed for live-imaging observation of MT dynamics during pollen development, root cell division, salt response, and cytoskeletal mutant characterization [224]. This research not only provides a valuable resource for studying the rice cytoskeleton but also demonstrates that species-specific MT marker design is necessary, especially for crop plants.

## 10. Conclusions

Recent progress in plant MT studies has highlighted the importance of MTs in basic plant biology research. The phenotypic diversity of plant organs, regulated by MTs via cell division and wall patterning, is critical for the successful marketing of a wide array of foods such as fruits, vegetables, seeds (grains), leaves, and tubers [226]. How plants use MT regulation to respond to multiple environmental signals to change their growth and development is an area of future research that could help make crop plants more resistant to climate change. With the discovery of more MAPs and the development of new techniques, our understanding of the mechanism of plant MT regulation will facilitate sustainable agriculture.

## Data Availability

This study did not generate any new data.
